# The concurrent stimulation of Wnt and FGF8 signaling induce differentiation of dental mesenchymal cells into odontoblast-like cells

**DOI:** 10.1007/s00795-021-00297-3

**Published:** 2021-11-05

**Authors:** Motoyoshi Kimura, Akiko Saito, Shoko Onodera, Takashi Nakamura, Makoto Suematsu, Seikou Shintani, Toshifumi Azuma

**Affiliations:** 1 Department of Pediatric Dentistry, Tokyo Dental College, 2-9-18 Kanda-Misaki-Chou, Chiyoda, Tokyo, 101-0061 Japan; 2 Department of Biochemistry, Tokyo Dental College, 2-9-18 Kanda-Misaki-Chou, Chiyoda, Tokyo, 101-0061 Japan; 3 Department of Biochemistry, Keio University School of Medicine, 35 Shinanomachi, Shinjukuku, Tokyo, 160-8582 Japan

**Keywords:** Dental mesenchymal cells, Canonical Wnt, FGF8, Odontoblast, Tooth development

## Abstract

Fibroblast growth factor 8 (FGF8) is known to be a potent stimulator of canonical Wnt/β-catenin activity, an essential factor for tooth development. In this study, we analyzed the effects of co-administration of FGF8 and a CHIR99021 (GSK3β inhibitor) on differentiation of dental mesenchymal cells into odontoblasts. Utilizing Cre-mediated EGFP reporter mice, dentin matrix protein 1 (Dmp1) expression was examined in mouse neonatal molar tooth germs. At birth, expression of Dmp1-EGFP was not found in mesenchymal cells but rather epithelial cells, after which Dmp1-positive cells gradually emerged in the mesenchymal area along with disappearance in the epithelial area. Primary cultured mesenchymal cells from neonatal tooth germ specimens showed loss of Dmp1-EGFP positive signals, whereas addition of Wnt3a or the CHIR99021 significantly regained Dmp1 positivity within approximately 2 weeks. Other odontoblast markers such as dentin sialophosphoprotein (Dspp) could not be clearly detected. Concurrent stimulation of primary cultured mesenchymal cells with the CHIR99021 and FGF8 resulted in significant upregulation of odonto/osteoblast proteins. Furthermore, increased expression levels of runt-related transcription factor 2 (Runx2), osterix, and osteocalcin were also observed. The present findings indicate that coordinated action of canonical Wnt/β-catenin and FGF8 signals is essential for odontoblast differentiation of tooth germs in mice.

## Introduction

Tooth development is initiated by thickening of oral epithelium and adjacent cranial neural crest (CNC) derived-mesenchyme, after which this epithelial-mesenchymal complex becomes invaginated into the underlying CNC to form a tooth bud. The mesenchyme then starts to express a specific set of transcription factors and signaling molecules, and gains an ability to induce tooth morphogenesis. Eventually, the epithelium differentiates into ameloblasts and mesenchyme into odontoblasts [1, 2]. The molecular regulation of early tooth morphogenesis leading to tooth bud formation has been extensively studied. Indeed, a large body of work shows that the network controlling tooth development includes major signaling pathways, such as those of transforming growth factor β (TGFβ), BMP, FGF, Wnt, and SHH, indicating recurrent functions at various stages [3, 4]. However, the precise molecular network controlling the late stages of tooth development including odontoblast differentiation is very complex and yet to be determined [5], and there is a need to provide more comprehensive understanding of odontoblast development.

Odontoblasts do not repair dentin in vivo despite their ability to create new dentin throughout life in response to damage by stem cells, termed dental pulp stem cells (DPSCs), which show similarities to human bone marrow stromal cells (BMSCs). DPSCs are capable of migrating to the dentin surface and differentiating into odontoblasts to form reparative dentin. However, unlike primary dentin, this reparative dentin is poorly organized, with irregular dentinal tubules embedded in the dentin matrix. It is also not yet known how primary dentin is organized by CNC-derived mesenchymal cells, while the genetic basis for odontogenic ability remains to be explained for dental epithelium and mesenchyme. The currently understood gene expression signature of dental mesenchyme during the early morphogenetic stage includes numerous potential candidates that might account for odontogenic competence of the tissue [6]. In particular, de novo tooth formation has been induced in transgenic mice by stimulating the canonical Wnt pathway, with constitutive expression of *β-catenin* in the ectoderm resulting in formation of extra teeth [7]. Therefore Wnt signaling, particularly canonical Wnt pathways, might have odontogenic inducing potential [8–10].

Several members of the FGF family are expressed during early development of the tooth germ and function at a definite stage of tooth development from the start to finish of tooth formation. Intensive FGF8 expression is initially detected in dental epithelium, presumably before onset of tooth formation, and then persists there until the early bud stage. FGF signaling is involved in limiting the site of tooth formation by inducing expressions of *Paired box gene 9* (*Pax9*), Paired-like homoedomain 1 (*Pitx1*), and Paired-like homoedomain 2 (*Pitx2*) [11]. FGF8 is involved in induction of BarH-like homeobox 1 (*Barx1*) in the expected intermolecular space [12], and also responds primarily to LIM/homeobox protein 6 (*Lhx6*) and LIM/homeobox protein 7 (*Lhx7*) expression in odontogenic mesenchyme before initiation of and during tooth formation [13]. However, it remains unclear whether FGF8 is a component of the suggestive odontogenic potential in oral epithelium, as in mice lacking FGF8 in the oral epithelium, most of the architecture including molars is used to form tooth germs, but not teeth. Another FGF, presumably FGF9, may rescue incisor formation in the absence of FGF8 [12]. It has also been speculated that FGF8 is involved in induction of FGF3 expression in tooth mesenchyme [14]. Therefore, though FGF8 is important for tooth development, its detailed mechanism is largely unknown. Presently, differentiation and maintenance of odontoblast cultures are difficult to perform, thus the effects of Wnt and FGF8 on odontoblast differentiation and maintenance remain to be elucidated.

In the present study, Dmp1-Cre-recombinase transgenic mice, developed by crossing with CAG-CAT-EGFP mice, were utilized as a reporter strain to detect Dmp1 expression in dental germ mesenchymal cells. In newly born mice, dental germ epithelial cells expressed Dmp1, while mesenchymal cells did not, though at 4 days after birth, dental germ mesenchymal cells began to express Dmp1. We also found that canonical Wnt signaling plays a key role in *Dmp1* expression in dental germ-derived mesenchymal cells. These findings suggest that the role of the Wnt canonical pathway is important in late stage odontogenesis.

Addition of FGF8 enhanced the degree of differentiation of tooth germ-derived mesenchymal cells into odontoblasts. Furthermore, some bone-related markers were increased, whereas others were decreased. An increase or decrease in *Runx2* may have effects on the border of differentiation into osteoblast/cyte or odontoblast. The present results show that interaction between the Wnt canonical pathway and FGF8 is important for odontoblast differentiation, providing new evidence for elucidation of the mechanism of tooth development.

## Materials and methods

### Animals

*Dmp1*-Cre mice were crossed with a transgenic mouse line carrying a reporter gene construct known as CAG-CAT-EGFP reporter mice [15] to obtain *Dmp1-EGFP* mice. All mice were anesthetized using isoflurane (Abbvie, North Chicago) before decapitation for tooth germ harvesting.

### Tissue preparation

Mandibles and first molars were taken from Dmp1-EGFP neonatal mice and fixed overnight in 4% paraformaldehyde phosphate buffer solution (Wako Pure Chemical Industries, Ltd., Osaka). Fixed tissues were then decalcified with 10% ethylenediaminetetraacetic acid (EDTA) (MUTO PURE CHEMICALS Co., Ltd., Tokyo) for 1 week. After decalcification, they were dehydrated with a series (10%, 20%, 30%) of sucrose (Sigma-Aldrich Co. LLC, St. Louis) and phosphate buffered saline (PBS) (Takara Bio Inc., Shiga). Tissues were embedded in optimal cutting temperature compound (O.C.T., Sakura Finetek Japan Co., Ltd., Tokyo) and 10-μm sections were obtained using a cryostat (Leica, Wetzlar). Fluorescent imaging was performed using a fluorescence microscopy system (BZ-X700) (KEYENCE Corp., Osaka).

### Cell cultures

Newborn mouse dental mesenchymal tissues were isolated from dental epithelium, then digested in 10% collagenase I (F. Hoffmann-La Roche, Ltd., Basel) for 2 h and 0.05% trypsin–EDTA (Thermo Fisher Scientific Inc., Waltham) for 8 min at 37 °C. Enzymatically digested tissues were washed three times with Dulbecco’s modified Eagle’s medium (DMEM) (Thermo Fisher Scientific Inc., Waltham) containing 10% fetal bovine serum (FBS) (Biosera Inc., Kansas City) and 1% penicillin streptomycin (Thermo Fisher Scientific Inc., Waltham). Murine dental mesenchymal cells were cultured in DMEM supplemented with 10% FBS and 1% penicillin streptomycin at 37 °C in a humidified atmosphere containing 5% CO_2_.

Cells were cultured in 12-well plates at a density of 1.0 × 10^4^ cells per well, then 50 ng/ml Wnt3a (R&D Systems, Inc., Minneapolis), 50 ng/ml Wnt5a (R&D Systems, Inc., Minneapolis), 25 ng/ml FGF8 (R&D Systems, Inc., Minneapolis), and 3 μM CHIR99021 (Sigma-Aldrich Co. LLC, St. Louis) were added, and culturing was continued for 3 weeks to induce differentiation. The concentration of each chemical regent was determined based on previous papers [16–19]. The medium, which contained cytokines, was changed every 2 days.

### Immunohistochemistry

Cells were fixed with 4% paraformaldehyde (Wako Pure Chemical Industries Ltd., Osaka) for 20 min at room temperature and rinsed three times with PBS. Fixed cells were permeabilized with 0.1% Triton X-100 (Wako Pure Chemical Industries, Ltd., Osaka) in PBS for 10 min. After membrane permeabilization, the cells were blocked with 5% goat serum (Wako Pure Chemical Industries, Ltd., Osaka) in PBS for 15 min, then incubated with primary antibodies, including monoclonal chicken antibody conjugated with anti-GFP IgG (Cell Signaling Technology, Inc., Danvers) and monoclonal rabbit antibody conjugated with anti-β-catenin IgG (Cell Signaling Technology, Inc., Danvers) at a dilution of 1:100 for 1 h at room temperature. After washing with cold 5% goat serum in PBS three times, the cells were treated with secondary goat anti-chicken Alexa Fluor 488 (Cell Signaling Technology, Inc., Danvers) or goat anti-rabbit Alexa Fluor 594 (Cell Signaling Technology, Inc., Danvers) at a dilution of 1:1000 for 1 h at room temperature. Fluorescent imaging was performed using a fluorescence microscopy system (BZ-X700) (KEYENCE Corp. Osaka).

### RNA isolation and quantitative PCR

Total RNA was isolated from each sample using QIAzol Lysis Reagent (Qiagen, Inc., Hilden) and RNA purity was assessed with a NanoDrop^®^ ND-1000 spectrophotometer (Thermo Fisher Scientific, Waltham, MA). cDNA was synthesized using a cDNA Reverse Transcription Kit (Thermo Fisher Scientific Inc., Waltham), according to the manufacturer’s instructions. qRT-PCR analysis was performed using Premix Ex Taq™ reagent (Takara Bio Inc., Otsu), according to the manufacturer’s protocol, and an Applied Biosystems^®^ 7500 Fast Real-Time PCR System. The primer sequences are listed in Table 1. Reactions were performed for 40 cycles of 5 s each at 95 °C and 34 cycles at 60 s. Relative mRNA expression was determined using the ΔΔCT method.

**Table 1:** Primers used for quantitative reverse transcription polymerase chain reaction (qRT-PCR)

Gene symbol	Left	Right	GenBank accession no
*β*-actin	ccaaccgtgaaaagatgacc	accagaggcatacagggaca	NM_007393.5
*Alp*	aatgaggtcacatccatcctg	cacccgagtggtagtcacaa	NM_007431.2
*β*-catenin	tgcagatcttggactggaca	aagaacggtagctgggatca	NM_001165902.1
*Bglap*	tgaggaccatctttctgctca	tggacatgaaggctttgtca	NM_007541.3
*Bsp*	gaaaatggagacggcgatag	cattgttttcctcttcgtttga	NM_008318.2
*Dmp1*	cgctgaggttttgaccttgt	ttgggatgcgattcctctac	NM_016779.2
*Dspp*	agccagtcagaagcatgtcc	cctttgttgggaccttcagt	NM_010080.3
*Nestin*	tcccttagtctggaagtggcta	ggtgtctgcaagcgagagtt	NM_016701.3
*Runx2*	cgtgtcagcaaagcttctttt	ggctcacgtcgctcatct	NM_001145920.2
*Panx3*	gaaatctctctggcctcacaa	atacatggccacagccaga	NM_172454.2
*Sp7*	ctcctgcaggcagtcctc	gggaagggtgggtagtcatt	NM_130458.3

### Flow cytometry analysis

Dental mesenchymal cells were harvested after culturing for 3 weeks using 0.05% trypsin and suspended in PBS containing 10% FBS. EGFP-positive cells were isolated using a FACSAria system (Becton, Dickinson and Company, Franklin Lakes).

### Statistical analysis

Quantitative values are expressed as the mean ± SD. One-way ANOVA was performed using the SPSS-Windows software package, v. 14.0 (SPSS Inc., Chicago). *P* values less than 0.01 were considered to indicate statistical significance.

## Results

### Dmp1-EGFP expression in conditional transgenic murine odontoblasts and ameloblasts

**Fig. 1:**
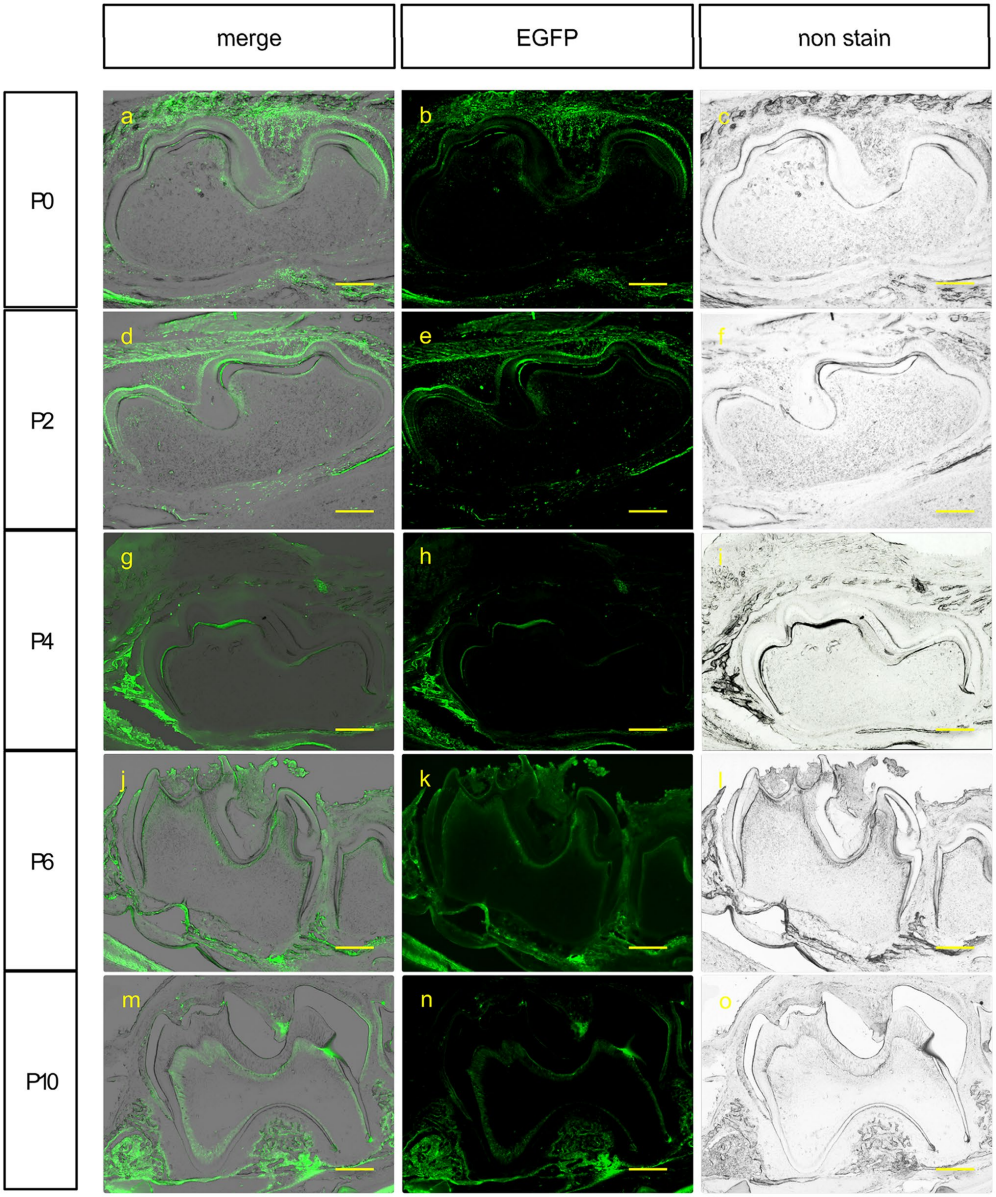
Evaluation of Dmp1-EGFP expression in conditional transgenic mice tooth germs. Photographs show M1 of mice immediately following birth and on days 2, 4, 6, and 10. Dmp1-EGFP expression was observed in postnatal tooth epithelium, which shifted to the mesenchyme with growth. The range of Dmp1-EGFP expression gradually expanded in the mesenchyme, while expression intensity in the tooth epithelium gradually decreased. Scale bar = 200 μm

There was nearly no EGFP expression detected in the dental mesenchyme area in newborn Dmp1-EGFP mice, while that was noted in ameloblast areas (Fig. 1a–c). Two days after birth, Dmp1-EGFP signals were observed in the layer where odontoblasts were located (Fig. 1d, e). Furthermore, we also observed that EGFP signals in alveolar bones became stronger, whereas Dmp1-EGFP signals gradually became weaker in ameloblasts (Fig. 1g–o).

### Activation of canonical Wnt signals and increased *Dmp1* expression

**Fig. 2:**
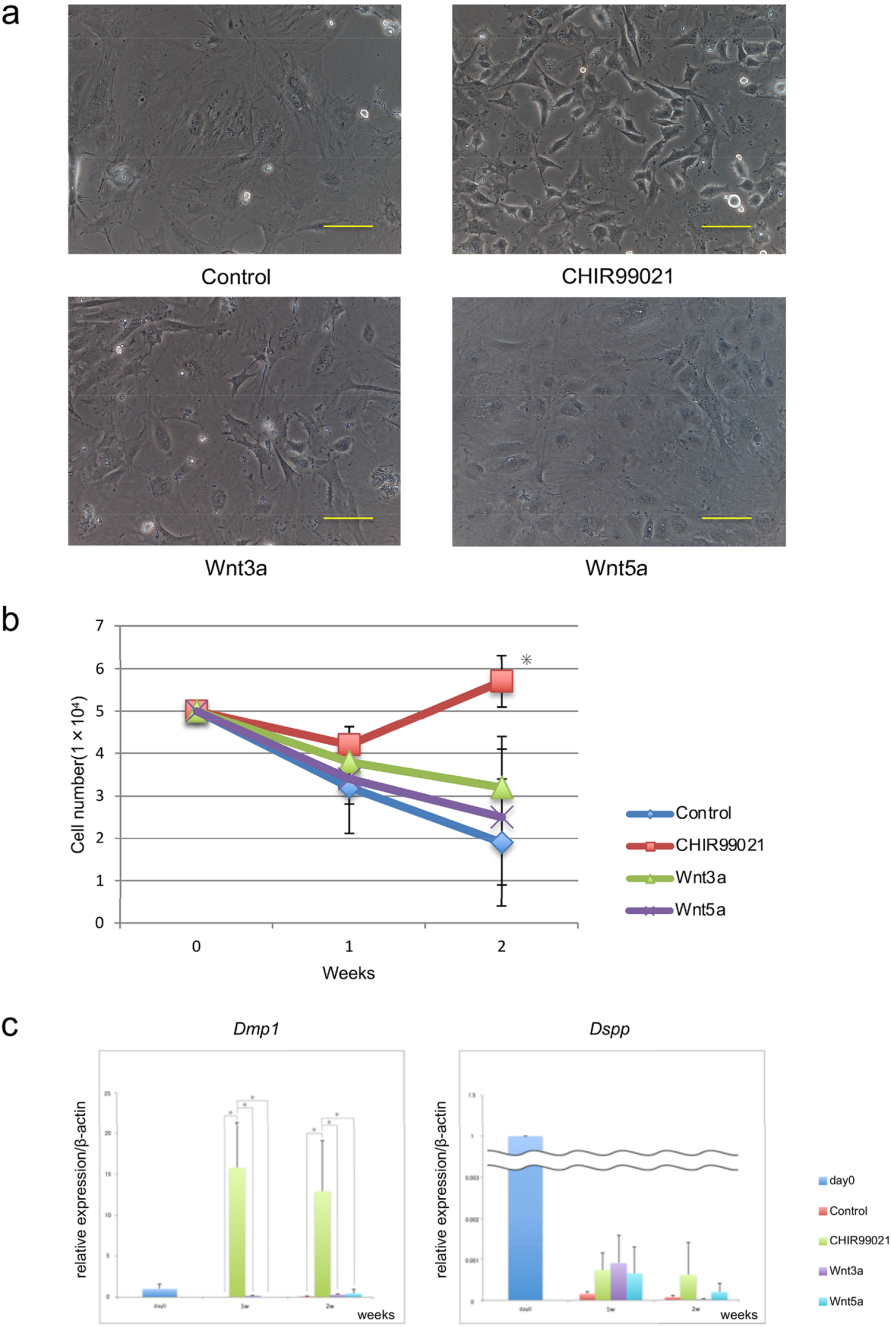
Activation of canonical Wnt signaling pathway increases osteogenic potential. **a** Morphology of tooth mesenchymal cells cultured with or without Wnt3a, Wnt5a, or CHIR99021. Morphological alterations were visible following administration of Wnt3a and especially CHIR99021. Cells cultured with CHIR99021 demonstrated a fibroblast-like spindle shape similar to that of odontoblast. Scale bar = 200 μm. **b** Growth curves of tooth mesenchymal cells with or without Wnt3a, Wnt5a, or CHIR99021. Values are shown as the mean ± SD from three independent experiments. *p < 0.01. **c** *Dmp1* expression was markedly increased following administration of the CHIR99021. On the other hand, *Dspp* expression was prominently decreased in all of the samples. *p < 0.01

**Fig. 3:**
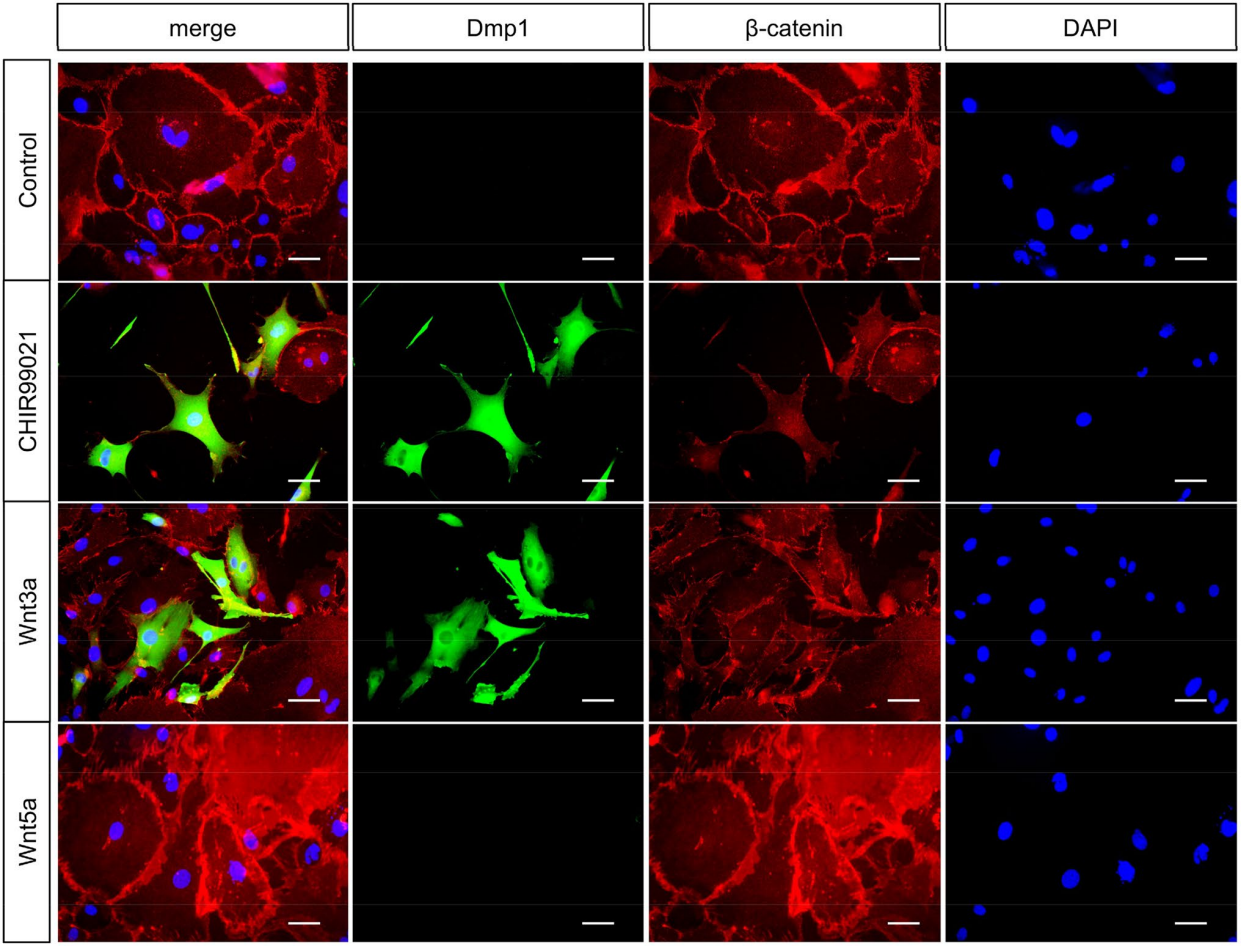
Promotion of β-catenin nuclear translocation by administration of CHIR99021. Cells were cultured in medium containing a Wnt signaling activator, and analysis of Dmp1-EGFP and β-catenin immunofluorescence was performed. Dmp1-EGFP expression was induced by activation of canonical Wnt signaling. In addition, β-catenin nuclear translocation was observed with CHIR99021 treatment. Scale bar = 50 μm

Wnt signals are thought to function as essential developmental modulators of ecto-mesodermal development. We investigated canonical and non-canonical Wnt signaling to determine the effects on odontoblastic differentiation. Primary cultured tooth germ mesenchymal cells were obtained from newborn Dmp1-EGFP mice. We observed no Dmp1-EGFP signals in tooth mesenchymal cells in newly born mouse tooth germ mesenchymal cells. Morphological alterations were visible following administration of Wnt3a and especially the following that of CHIR99021, while cells cultured with the latter demonstrated a fibroblast-like spindle shape similar to that of odontoblasts. Administration of Wnt5a to activate the non-canonical Wnt pathway caused no obvious changes as compared to the control (Fig. 2a). Cells treated with CHIR99021 showed greater cell proliferation as compared with Wnt3a- or Wnt5a-treated cells (Fig. 2b). Following treatment with Wnt3a, CHIR99021, and Wnt5a, *Dmp1* expression was remarkably induced by CHIR99021. Also, *Dspp* expression was substantial in day 0 cultured cells, but was remarkably reduced to an undetectable level soon after beginning culturing. On the other hand, administrations of CHIR99021 and Wnt3a were able to maintain *Dspp* expression to some extent in cultured tooth germ mesenchymal cells (Fig. 2c). Immunofluorescence microscopy showed that administrations of CHIR99021 and Wnt3a induced Dmp1-EGFP expression, especially CHIR99021 treatment, whereas Wnt5a failed to induce that. Nuclear β-catenin localization indicating Dmp1-EGFP expression was also found to be induced by canonical Wnt signaling (Fig. 3).

### Differentiation into odontoblasts by CHIR99021 and FGF8 concurrent treatment

**Fig. 4:**
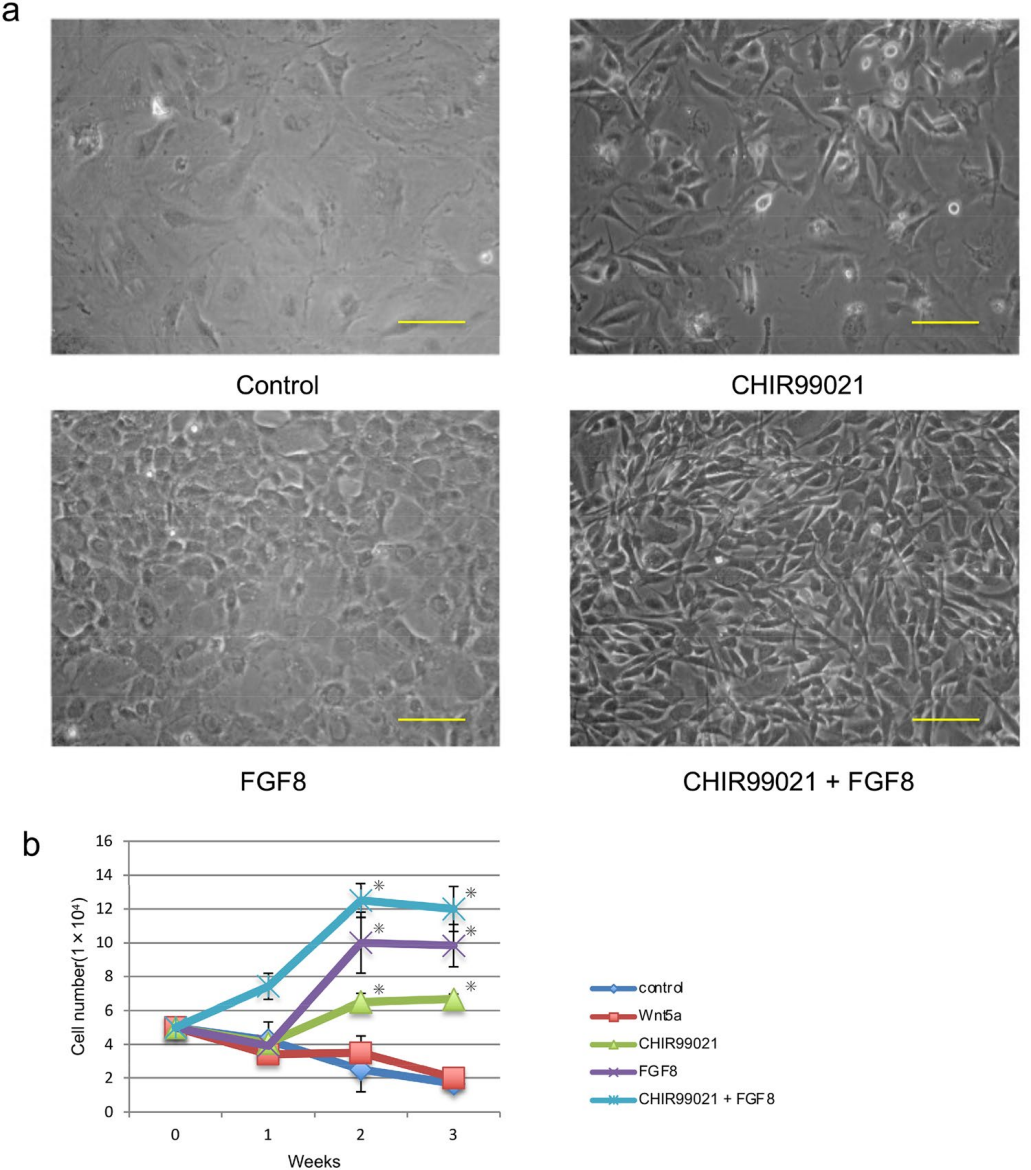
Administration of CHIR99021 with or without FGF8. **a** Photographs show cells cultured with a single administration of the CHIR99021 or FGF8, as well as both for 3 weeks. Cells with both the CHIR99021 and FGF8 added exhibited a spindle shape. **b** Comparison of cell growth curves with single administration of CHIR99021 or FGF8, and both. The values are shown as mean ± SD from three independent experiments. *p < 0.01

**Fig. 5:**
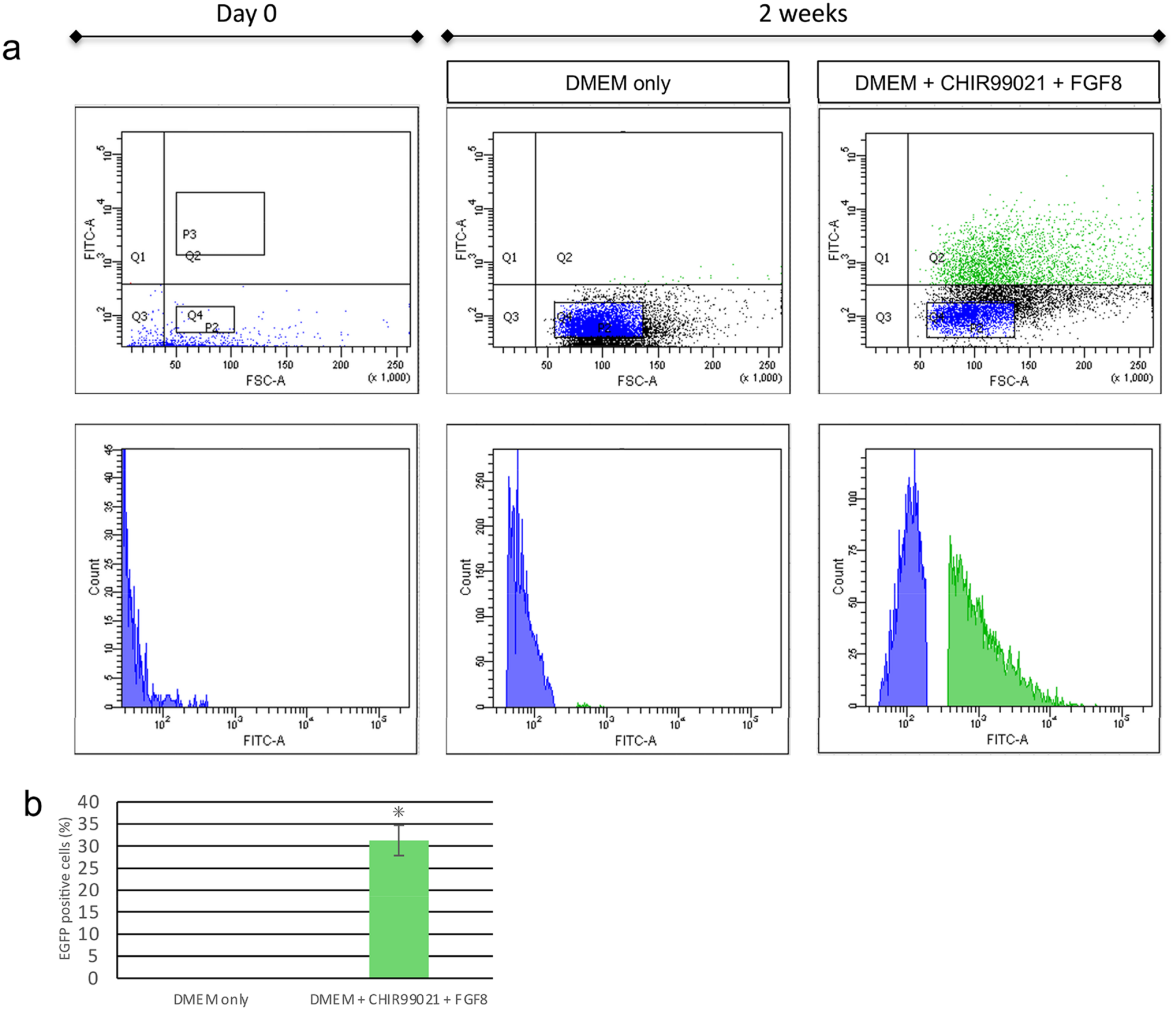
Isolation of cultured cells using flow cytometry. **a** Addition of the CHIR99021 with FGF8 induced Dmp1-EGFP expression in cells subjected to flow cytometry analysis. Upper graph: vertical axis indicates EGFP fluorescence intensity and the horizontal axis cell size. Lower graph: vertical axis indicates cell number and the horizontal axis cell size. Blue, Dmp1-EGFP negative cells. Green, Dmp1-EGFP positive cells. **b** Quantification of Dmp1-EGFP positive cells by flow cytometry. Cells were cultured with or without CHIR99021 and FGF8 as indicated. Blue, Dmp1-EGFP negative cells. Green, Dmp1-EGFP positive cells. *p < 0.01

While *Dmp1* expression was induced by CHIR99021 treatment of tooth germ mesenchymal cells, *Dspp* expression was sharply decreased after primary culturing of tooth germ mesenchymal cells, thus we concurrently treated the cells with CHIR99021 and FGF8. Phase imaging showed that FGF8 caused cell growth and the cell morphology had a cobblestone-like appearance, while CHIR99021 treatment induced a spindle-like morphology. Interestingly, simultaneous treatment with both FGF8 and CHIR99021 resulted in a spindle-like shape similar to that of odontoblasts (Fig. 4a). In addition, cells with both FGF8 and CHIR99021 added showed greater cell proliferation as compared to those with a single administration (Fig. 4b). Furthermore, flow cytometric analysis showed that tooth mesenchymal cells treated with both CHIR99021 and FGF8 induced differentiation to Dmp1-EGFP-positive cells (Fig. 5a), and the number of Dmp1-EGFP-positive cells were significant upregulated following treatment with both as compared to the non-treated group. (Fig. 5b).

**Fig. 6:**
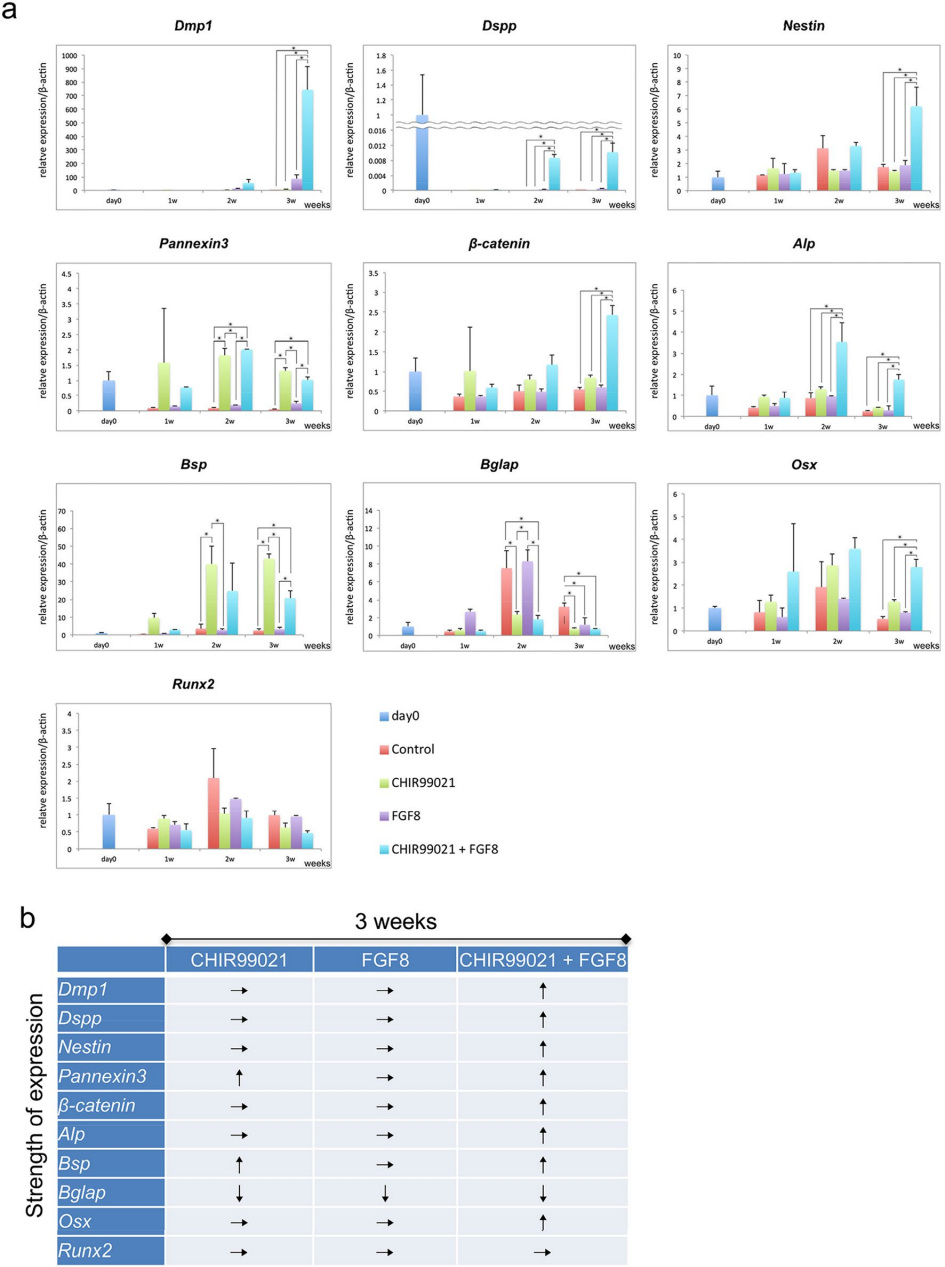
Evaluation of odontoblast differentiation as effects of CHIR99021 and FGF8. **a** Shown are mRNA levels of dental osteo/odonto-related genes in freshly isolated mDMCs (day 0) as well as mDMCs cultured in CHIR99021-, FGF8-, and CHIR99021/FGF8-supplemented medium. *p < 0.01. **b** mRNA expression schema of examined genes after 3 weeks. Arrows indicate expression intensity of each sample as compared to control

qRT-PCR analyses revealed remarkable levels of expression of Dmp1, Dspp, Nestin, Pannexin3, Bsp, and Osterix (Osx) following treatment with CHIR99021 and FGF8 as compared with either administered alone. Also, treatment with CHIR99021 alone caused an increase in expression of Pannexin3 and Bone sialoprotein (Bsp), indicating promotion of canonical Wnt pathway activation. On the other hand, no increase in expression of any of these factors was seen with FGF8 alone (Fig. 6a, b). Together, these results indicate that canonical Wnt activation along with FGF8 significantly induces odontogenesis of tooth germ mesenchymal cells.

## Discussion

In the present study, three main results were obtained. First, neonatal molar mesenchymal cells lost the odontoblastic phenotype immediately after cultivation. Second, canonical Wnt/β-catenin signaling had some positive effects on odontoblast differentiation. Third, simultaneous treatment with canonical CHIR99021 and FGF8 gave rise to promotion of odontoblastic differentiation.

Although several reports have been presented indicating that mesenchymal odontoblastic differentiation relies nearly entirely on signals from adjacent epithelial cells [4, 9, 20–22], little is known about the molecular events that cause mesenchymal stem cells to proliferate and differentiate into odontoblast-like cells [23]. Thus, the main points of investigation in this study were determination of signals that induce mesenchymal odontoblast genesis and how those signals function.

The canonical Wnt/β-catenin signaling pathway is important for stem cell renewal, proliferation, and differentiation [24, 25]. During tooth development, Wnt/β-catenin signaling is required at various stages of tooth morphogenesis. In the canonical Wnt / βcatenin signaling pathway, gene expression is regulated by expression of *β-catenin* in the cytoplasm, and Wnt1, Wnt2, Wnt3, Wnt3a, Wnt7a are the main ligands. Inactivation of *β-catenin* expression during tooth development leads to disruption of odontoblast differentiation and root formation, whereas its overexpression results in excessive dentin production from mature odontoblasts, indicating that canonical Wnt/β-catenin signaling plays important roles in odontoblast development and maturation [26, 27]. We treated mouse neonatal tooth germ mesenchymal cells with CHIR99021, a specific and reversible inhibitor of GSK3β activity. Inhibition of GSK3β by CHIR99021 has been shown to result in activation of the Wnt signaling pathway, as well as sustained pluripotency of human and mouse embryonic stem cells [16]. We found that CHIR99021 treatment induced expression of Dmp1, an odontoblast marker protein. The Cre-LoxP reporter system was utilized to detect Dmp1 expression and that was found in neonatal primary cultured mesenchymal cells from the tooth germ, which were observed to be negative for Dmp1-EGFP expression, as that was not observed until 2 days after birth. Primary cultured tooth germ mesenchymal cells were successfully established and Wnt3a treatment was found to have some effect to induce EGFP expression linked with Dmp1 expression, whereas Wnt5a did not. Expression of Wnt3a is reported in Hertwig’s epithelial root sheath of the 2-week-old mice, but not enough research in the early stages of tooth germ formation. These observations suggested that canonical Wnt/β-catenin signaling positively supports odontoblast genesis by Wnt3a. In previous studies, we investigated *Dspp*, another important odontoblast marker [28, 29], and found only a modest increase in expression, indicating that canonical Wnt/β-catenin signaling was not enough to induce odontoblast genesis. Other reports have noted that the Wnt/β-catenin pathway has a positive effect on *Runx2* expression by osteoblasts and drives maturation [30, 31]. Thesleff et al. reported that canonical Wnt signaling induced Runx2 expression in odontoblasts, while *Runx2* appeared to inhibit differentiation [32]. Together with those previous reports, the present results indicate that canonical Wnt/β-catenin can sustain *Runx2* expression expressed by both odontoblasts and osteoblasts, which in turn is speculated to suppress the odontoblast-specific marker protein *Dspp*. Wnt has at least two intracellular signaling pathways named canonical and non-canonical pathway. Wnt5a is one of the ligands for the non-canonical pathway, while Wnt3a is one of those for the canonical pathway. Wnt5a regulates cell proliferation, migration, and polarization [33], and the non-canonical Wnt pathway has been shown to inhibit canonical Wnt/β-catenin signaling by promoting degradation of β-catenin in a GSK3-independent manner [34]. CHIR99021 is a small molecular compound that inhibits β-catenin, a downstream component of the canonical Wnt pathway. The present results indicate that when Wnt3a has a tendency to induce odontogenic differentiation. Furthermore, CHIR99021 showed significant upregulation though Wnt5a did not. In addition, it was demonstrated that activation of the canonical Wnt pathway is important for inducing odontogenic differentiation. Therefore, it is speculated that CHIR99021, which is less susceptible to negative feedback than cytokines, induces stronger dental differentiation.

FGF8 in the oral ectoderm defines tooth formation sites by activating expression of the dental mesenchymal marker *Pax9* and has been reported to be a pro-odontogenic factor [11], thus we considered that it may also be an early odontogenic specifier. The present results showed that FGF8 had positive effects on proliferation as well as *Dspp* expression, while it also induced a reduced level of Runx2 expression, which likely has positive effects on odontoblastic differentiation. The functions of FGF8 are diverse, including mitogenic/proliferative, as well as regulatory morphological effects. As shown in Fig. 4, FGF8 treatment alone induced a cobblestone-like appearance of tooth germ mesenchymal cells, and those treated with both CHIR99021 and FGF8 showed significantly enhanced growth and a spindle-like morphology. In addition to morphological and proliferative effects, significant increases in osteogenic markers *Dmp1, β-catenin, Alp* and *Osx,* were also observed. Also, combined treatment with CHIR99021 and FGF8 resulted in remarkable increases in odontogenic-specific *Dmp1, Dspp, Nestin*, and *Pannexin3*. Wnt, FGF, and BMP are known to be interconnected for dental development, and *FGF9* and *FGF10* are also associated with the Wnt pathway during odontogenesis [35]. However, that study only found co-localization or canonical Wnt pathway enhancement of FGF expression. The present report is the first to show that simultaneous treatment with FGF8 and CHIR99021 significantly enhances odontoblastic differentiation. Our results also indicate that this combination treatment has preferential effects on odontoblast genesis, as that produced different changes in osteoblast markers as well as increased the expression of all odontoblast markers.

## Conclusion

In summary, *Dmp1* was found to have a well-differentiated expression in odontoblasts. Pre-matured tooth germ mesenchymal cells expressed neither *Dmp1* nor *Dspp*. It was suggested that concurrent stimulation of Wnt and FGF8 signaling induces differentiation of dental mesenchymal cells into odontoblast-like cells. Although additional detailed investigations are needed, the present observations provide important cues for a more detailed elucidation of odontoblast genesis.
